# *MET*基因改变的非小细胞肺癌靶向治疗耐药机制及治疗策略

**DOI:** 10.3779/j.issn.1009-3419.2023.102.33

**Published:** 2023-09-20

**Authors:** Shuzhan LI, Xinwei ZHANG

**Affiliations:** 300060 天津，天津医科大学肿瘤医院生物治疗科，国家恶性肿瘤临床医学研究中心，天津市“肿瘤防治”重点实验室，天津市恶性肿瘤临床医学研究中心，天津市肿瘤免疫与生物治疗重点实验室; Department of Immunology, Tianjin Medical University Cancer Institute and Hospital, National Clinical Research Center for Cancer, Tianjin Key Laboratory of Cancer Prevention and Therapy, Tianjin's Clinical Research Center for Cancer, Key Laboratory of Cancer Immunology and Biotherapy, Tianjin 300060, China

**Keywords:** 肺肿瘤, 间质表皮转化因子, 靶向治疗, 耐药, 治疗策略, Lung neoplasms, Mesenchymal to epithelial transition factor, Targeted therapy, Drug resistance, Treatment strategy

## Abstract

间质表皮转化因子（mesenchymal to epithelial transition factor, MET）基因改变参与了非小细胞肺癌的增殖、侵袭和转移。MET-酪氨酸激酶抑制剂（tyrosine kinase inhibitors, TKIs）已获批用于MET基因改变的非小细胞肺癌，而这些药物的耐药不可避免。MET-TKIs的分子耐药机制错综复杂，尚不完全清楚。本文主要对这些MET-TKIs的潜在耐药机制进行综述，以期为MET基因改变的患者提供合理的治疗思路。

## Abstract (en)

Mesenchymal to epithelial transition factor (MET) gene alterations involve in the proliferation, invasion, and metastasis of non-small cell lung cancer. MET-tyrosine kinase inhibitors (TKIs) have been approved to treat non-small cell lung cancer with MET alterations, and resistance to these TKIs is inevitable. Molecular mechanisms of resistance to MET-TKIs are completely unclear. The review focused on potential mechanisms of MET-TKIs resistance and therapeutics strategies to delay and prevent resistance.

近年来多个靶向药物获批用于治疗非小细胞肺癌（non-small cell lung cancer, NSCLC），大大延长了患者的生存^[1]^。间质表皮转化因子（mesenchymal to epithelial transition factor, MET）作为NSCLC的一个重要驱动基因，存在MET基因14号外显子跳跃突变（MET exon 14 skipping mutation, METex14）、MET扩增、过表达、融合和错义突变等多种活化形式^[2]^。目前多种MET抑制剂获批上市，其发生耐药不可避免，本文将阐述MET酪氨酸激酶受体抑制剂（MET tyrosine kinase inhibitors, MET-TKIs）耐药的机制，并分析其临床对策。

## 1 MET分子及NSCLC中MET的突变方式

MET属于跨膜受体酪氨酸激酶家族，其基因位于染色体7q21-q31，长度约为125 kb，含有21个外显子^[3]^。MET与其配体肝细胞生长因子（hepatocyte growth factor, HGF）结合后，形成同源二聚体，使胞内区（Y1234/1235、Y1349/1356）发生磷酸化^[4]^，通过SH2结构域招募下游多种效应分子，进而激活细胞外信号调节激酶/丝裂原活化蛋白激酶（extracellular signal-regulated kinase/mitogen activated protein kinase, ERK/MAPK）、磷脂酰肌醇3-激酶/丝氨酸-苏氨酸蛋白激酶B（phosphatidylinositol 3-kinase/protein kinase B, PI3K/AKT）、酪氨酸激酶/信号传导子和转录激活子（Janus kinase/signal transducer and activator of transcription, JAK/STAT）和核因子κB（nuclear factor kappa B, NF-κB）等信号通路，调控细胞增殖迁移与浸润、上皮间质转化、血管生成、抗凋亡、细胞干性维持等过程^[5]^。MET通路存在多种负向调节方式，包括位于14号外显子的Y1003残基介导的泛素化降解^[6]^、蛋白激酶C（protein kinase C, PKC）介导的S985残基磷酸化负向调节MET活性^[7]^、Ca^2+^-丝氨酸激酶相关的负调节机制^[8]^等。

MET作为癌基因，存在多种变异，参与恶性肿瘤的发生^[9]^。在NSCLC中，METex14导致MET基因转录后的14号外显子被错误剪接，致使MET稳定性增加并持续激活^[10]^。METex14在肺腺癌中发生率为3%-4%^[11,12]^，在较罕见的肉瘤样肺癌中发生率较高，为13%-22%^[13⇓-15]^。MET扩增（MET amplification, METamp）包括多倍体形成和基因局部扩增两种方式，其中局部扩增具有更高的HGF配体非依赖性^[16]^。METamp在原发性变异中发生率为2%-5%^[17]^，在表皮生长因子受体-酪氨酸激酶抑制剂（epidermal growth factor receptor-tyrosine kinase inhibitors, EGFR-TKIs）继发性耐药中发生率较高，为5%-20%^[18,19]^。MET蛋白过表达可由METex14、METamp等多种基因变异引起^[20]^，在NSCLC发生率为35%-72%^[17,21]^。上述METex14、METamp以及MET过表达均已证实对MET-TKIs敏感。而MET融合（MET fusion）及MET酪氨酸激酶结构域（MET-tyrosine kinase domain, MET-TKD）突变在NSCLC中更为罕见，其中MET融合突变均为个案报道，如MET-KIF5B^[22]^、MET-STARD3NL^[23]^、MET-HLA-DRB1^[24]^、MET-ATXN7L1^[25]^，均对克唑替尼（Crizotinib）治疗产生应答。MET-TKD突变在初治NSCLC患者中比例为0.06%，其中占比最多的为H1094Y^[26,27]^。

## 2 MET-TKIs

目前已获批用于肺癌的MET-TKIs依其作用机制可分为：I和II类。I类TKIs结合于MET催化结构域；II类TKIs结合于MET调节性结构域^[28,29]^。

### 2.1 I类MET-TKIs

目前获批用于METex14突变的NSCLC抑制剂均为I类MET抑制剂，通过与MET活化环中的Y1230结合，占用ATP结合袋抑制MET活性。Ia类与MET分子为非特异性结合，代表药物为克唑替尼（Crizotinib）。Ib类为MET高选择性抑制剂，有卡马替尼（Capmatinib）、特泊替尼（Tepotinib）、赛沃替尼（Savolitinib）与谷美替尼（Gumarontinib）等^[30,31]^。

在一项II期GEOMETRY mono-1多中心研究^[32]^中，卡马替尼在初治METex14阳性的晚期NSCLC患者中客观缓解率（objective response rate, ORR）达68%，中位缓解持续时间（median duration of response, mDoR）为12.6个月，中位无进展生存期（median progression-free survival, mPFS）为12.4个月，在经治患者中ORR为41%。而在METamp（拷贝数≥10）初治NSCLC患者中的ORR为40%，在经治患者中ORR为29% 。VISION研究^[33]^中，特泊替尼在METex14阳性的初治或经治NSCLC患者中的ORR为45%，mDoR为11.1个月，mPFS为8.9个月，且安全可耐受。赛沃替尼是我国首个获批的MET抑制剂，在II期临床研究（NCT02897479）^[34]^中，赛沃替尼治疗METex14阳性的不可切除或转移性肺肉瘤样癌或其他类型NSCLC的ORR为49.2%，且表现出良好的安全性和耐受性。谷美替尼于2023年3月8日获批上市，用于治疗具有METex14突变的局部晚期或转移性NSCLC。在GLORY研究^[31]^中，整体患者的ORR为66%，mPFS为8.5个月，在初治和经治患者中分别为11.7和7.6个月。

Ia类代表药物克唑替尼结合MET溶剂前沿的G1163残基发挥作用，G1163R突变可导致克唑替尼耐药，对Ib类抑制剂敏感，这也是克唑替尼疗效逊于Ib类MET-TKIs的原因之一。克唑替尼目前批准用于治疗间变性淋巴瘤激酶（anaplastic lymphoma kinase, ALK）、c-ros肉瘤致癌因子-受体酪氨酸激酶（ROS proto-oncogene 1, receptor tyrosine kinase, ROS1）突变阳性的局部晚期或转移性NSCLC。在PROFILE 1001临床研究^[35]^中，克唑替尼用于METex14阳性的NSCLC患者ORR为32%，mDoR为9.1个月，mPFS为7.3个月，疗效低于高选择性MET-TKIs。同样，克唑替尼对于METamp患者的疗效也非常有限^[36,37]^。

### 2.2 II类MET-TKIs

相较于Ib类MET-TKIs的高选择性，II类TKIs为ATP竞争性的、多靶点的小分子TKIs^[30]^，包括卡博替尼（Cabozantinib）、美乐替尼（Merestinib）、格来替尼（Glesatinib）和福瑞替尼（Foretinib）。卡博替尼为多靶点广谱TKIs，作用位点包括MET、血管内皮生长因子受体（vascular endothelial growth factor receptor, VEGFR）、ROS1、RET等，对METex14患者有一定疗效^[38]^。美乐替尼（LY2801653）作用靶点包括MET、RON、FLT3、ROS1、MERTK、AXL等多个受体酪氨酸激酶，II期临床研究正在进行（NCT02920996）。格来替尼（MGCD265）是MET、AXL、血小板衍生生长因子受体（platelet-derived growth factor receptor, PDGFR）家族等多靶点的抑制剂，已在I期临床研究中证实针对MET变异NSCLC患者中的ORR为30.0%^[39]^。福瑞替尼（GSK1363089）是MET、AXL、VEGFR等多靶点的抑制剂，在携带胚系MET突变的晚期乳头状肾癌中的ORR达到50%^[40]^，在NSCLC中的疗效尚待证实。

## 3 MET-TKIs耐药机制

近年来多个MET-TKIs研发上市，使许多MET基因改变的NSCLC患者获得生存获益，但在治疗中仍会发生原发性和继发性耐药。原发性耐药通常指MET改变的患者对MET-TKIs无法产生初始治疗应答，在卡马替尼、特泊替尼和赛沃替尼治疗中的发生率为4%-19.2%^[32,34,41]^。一项回顾性研究^[42]^发现，65例METex14阳性NSCLC的耐药患者，3例在治疗前即存在PIK3CA共突变，均在初次疗效评价时出现疾病进展。因此推测，旁路或下游途径存在激活变异是导致原发性耐药的主要机制。继发性耐药通常表现为患者对MET-TKIs治疗产生治疗应答数月或数年后出现进展，目前被认为是TKIs治疗的最大挑战。按照发生机制的不同，继发性耐药通常被分为MET依赖型耐药与非MET依赖型耐药（图1）。MET依赖型约占1/3^[26]^，包括MET的二次突变、MET基因扩增等。非MET依赖型耐药指MET下游信号的活化和旁路途径的激活等。

**图1:**
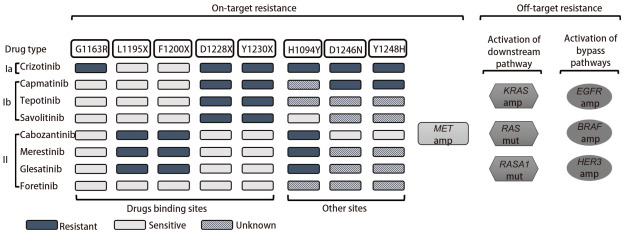
MET-TKIs的获得性耐药分为MET依赖型耐药机制与非MET依赖型耐药机制。MET依赖型耐药包括MET分子的二次点突变与MET扩增。非MET依赖型耐药包括了MET下游通路与旁路信号通路的激活。谷美替尼因上市时间短无相关报道。

### 3.1 MET依赖型耐药机制

已知MET D1228、Y1230、G1163、L1195、F1200等位点的突变以及MET基因扩增，参与了MET-TKIs的获得性耐药。这些突变位点常参与MET-TKIs与MET分子的结合。Ib类高选择性MET-TKIs的主要耐药突变位点为D1228X与Y1230X^[43]^。其中Y1230是Ib类TKIs与MET结合的重要氨基酸残基，而D1228参与MET激酶与ATP的结合^[44]^。Ia类药物克唑替尼耐药突变包括了D1228X与Y1230X^[43]^。克唑替尼同时需要与溶剂前沿残基G1163结合发挥抑制作用^[44]^，因此克唑替尼的耐药突变还包括G1163X^[43]^。

II类MET-TKIs的耐药突变有L1195X和F1200X^[43]^。II类MET-TKIs与非活化的MET分子的结合，位于ATP结合疏水口袋附近的L1195与F1200残基发挥了重要作用^[28,43]^。

在临床实践中，建议MET-TKIs耐药后进行二次活检及第二代测序技术（next-generation sequencing, NGS）检测，了解耐药机制，寻找个体化的治疗方案。Recondo等^[26]^报道了3例携带METex14的NSCLC患者应用克唑替尼耐药后分别检测出D1228H、D1228N、Y1230C突变，另有1例患者应用克唑替尼耐药后在血浆中检测出G1163R、D1228H/N、Y1230H/S、L1195V共突变。1例患者应用卡马替尼耐药后出现D1228N突变。除上述耐药突变位点外，H1094Y、Y1248H、D1246N也可能参与了MET依赖型耐药。1例应用格来替尼进展后出现H1094Y、L1195V共突变。2例MET过表达的NSCLC患者分别应用克唑替尼和卡马替尼，进展后分别出现Y1248H、D1246N突变^[45]^。

在体外研究^[28,43]^中，已证实的耐药突变还包括G1090A（I类耐药）、V1092I/L（Ia类耐药）、D1133V（II类耐药）。MET 14号外显子等位基因扩增也是MET-TKIs的潜在耐药机制。1例METamp患者应用格来替尼后出现17个MET 14号外显子等位突变，更换为克唑替尼后MET扩增至47个^[26]^。

MET基因改变是EGFR-TKIs治疗的耐药机制之一，MET-TKIs与EGFR-TKIs联合治疗可作为二线方案。一项回顾性研究^[27]^，对20例二线应用MET-TKIs联合EGFR-TKIs治疗的NSCLC患者出现耐药后再次进行NGS，发现L1195V（4例）、D1228H（8例）/N（15例）/Y（2例）、Y1230C（2例）/H（3例）、ALK融合（1例）、BRAF突变（3例），其中部分为共突变，另有1例出现D1228_M1229delinFL突变，推测MET-TKIs作为二线治疗的耐药机制更为复杂，可能由多种机制共同构成，尚需进一步研究证实。

### 3.2 非MET依赖型耐药机制

非MET依赖型耐药机制主要指不依赖于MET分子活化而导致的信号转导异常，可大致分为MET下游通路的异常激活（如与RAS/MAPK通路、PI3K/AKT通路有关的变异）和旁路途径的过度激活[如EGFR、靶向人表皮生长因子受体2/3（human epidermal growth factor receptor 2/3, HER2/3）、鼠类肉瘤病毒癌基因同源物B1（v-raf murine sar-coma viral oncogene homolog B1, BRAF）的扩增激活]。在20例经MET-TKIs治疗的METex14阳性NSCLC患者中，有9例（45%）分别出现KRAS扩增、KRAS点突变以及EGFR、HER3、BRAF扩增^[26]^。Guo等^[46]^报道了33%（5/15）经MET-TKIs治疗的患者发生MET非依赖性耐药，包括了KRAS扩增和点突变、RASA1突变以及EGFR扩增。目前针对MET非依赖型耐药机制的研究主要通过对MET-TKIs耐药患者的血液或组织学样本进行DNA或RNA水平检测来进行，因此表观遗传水平、蛋白表达水平的变异尚不能明确，尚有25%-47%的患者无法明确耐药机制^[26,46]^。Ia类克唑替尼应用于ALK融合突变阳性的NSCLC患者后可能出现P-糖蛋白（P-glycoprotein, P-gp）过表达，导致药物转运异常产生耐药^[47,48]^。因此推测药物转运异常也是MET-TKIs耐药机制之一。组织学类型转化是EGFR-TKIs治疗后耐药的机制之一^[49]^，目前尚未有MET-TKIs治疗耐药后出现组织学类型转化的病例报道。

## 4 MET-TKIs耐药后治疗策略

发生MET-TKIs耐药后，通过二次活检及NGS明确耐药机制，是选择后续治疗方案的重要依据。对于NGS可明确的耐药机制，可寻求靶向治疗或联合靶向治疗。对于机制未明的疾病进展，则需要进行其他局部治疗或全身治疗。下面分别从靶向治疗策略及其他治疗策略讨论MET-TKIs耐药后的治疗选择。

### 4.1 靶向治疗策略

 20%-35%的患者进行耐药后分子诊断可能发现MET依赖型耐药机制^[26,46]^。其中，约有1/3的MET依赖型机制导致的耐药可通过I/II类MET-TKIs序贯治疗解决，但临床证据有限^[26,50,51]^。如前所述，I/II类MET-TKIs诱导的耐药突变发生于不同位点，为I/II类MET-TKIs序贯治疗提供了分子基础^[43]^。I/II类MET-TKIs序贯应用可克服耐药首先在2017年被提出^[44]^。而后许多研究^[43,52,53]^通过体外或体内研究证实了序贯治疗的有效性。D1228H/N、Y1230C/H/S为常见Ib类MET-TKIs的特征性耐药突变，可尝试II类MET-TKIs治疗。G1163R为Ia类克唑替尼的特征性耐药突变，对Ib类（卡马替尼、赛沃替尼）和II类（美乐替尼）MET-TKIs为敏感突变^[43]^。对于II类MET-TKIs的耐药突变L1195F/V与F1200I/L，换用I类MET-TKIs可抑制肿瘤生长^[43,52]^。在一项II期临床研究^[54]^中，部分克唑替尼治疗后的METex14阳性NSCLC患者换用卡马替尼治疗后有一定临床获益，ORR为13%（2/15），疾病控制率（disease control rate, DCR）为80%（12/15）。Bahcall等^[50]^报道了1例MET D1228V导致的克唑替尼耐药，在应用卡博替尼后再次获得缓解。Klempner等^[55]^报道了携带METex14的NSCLC患者应用克唑替尼进展后换用卡博替尼后继续获益。

对于Y1248H、D1246N、H1094Y突变，也有体内外研究证实序贯治疗的有效性。Y1248H、D1246N分别出现在I类克唑替尼、卡马替尼治疗后，体外实验^[45]^证实其对II类TKIs敏感。1例具有METex14合并MET C526F突变的NSCLC患者应用克唑替尼进展后D1246N突变，更换为卡博替尼后再次达到部分缓解^[56]^。H1094Y产生于II类MET-TKIs格来替尼治疗后，并经体外实验证实对Ib类赛沃替尼敏感^[26]^。

尚有部分MET依赖型耐药不能通过序贯治疗克服。如D1228A/Y在体外研究^[43]^中证实对I/II类MET-TKIs均耐药。耐药共突变，如D1228H/N、Y1230H/S与L1195V和G1163R共突变在1例克唑替尼耐药患者的血浆样本中检测到^[26]^，对治疗选择提出新挑战。对于MET扩增导致MET-TKIs耐药的患者，序贯MET-TKIs治疗无效^[26]^。对于上述耐药机制，也可尝试MET单抗或EGFR/c-MET双特异性抗体。在I期CHRYSTALIS研究^[57]^中，19例经MET-TKIs治疗后的NSCLC患者应用埃万妥单抗（Amivantamab）治疗，ORR为21%（4/19），临床获益率为57.9%（11/19）。MET单抗Sym-015用于既往应用过MET-TKIs的NSCLC患者DCR为60%，mPFS为5.4个月^[58]^。

非MET依赖型耐药机制可通过MET-TKIs与靶向其他靶点的药物联合应用来克服。对于下游RAS/MAPK通路激活，体外实验^[59]^已经证实，MET-TKIs联合MEK抑制剂能够抑制携带METex14-KRAS G12D共突变细胞的生长。对于下游PI3K/AKT通路的激活，临床前研究^[42]^已证实联合应用MET-TKIs与PI3K抑制剂的有效性。旁路途径激活，如EGFR扩增、HER2扩增、BRAF扩增，同样可通过靶向联合治疗克服。对于EGFR扩增导致的MET-TKIs耐药，EGFR/c-MET双特异性抗体埃万妥单抗具有一定疗效^[60⇓-62]^。针对HER2扩增或BRAF扩增，联合应用MET-TKIs与相应靶向药物理论上能够产生疗效。

MET抗体偶联药物（antibody-drug conjugate, ADC）类药物已进入临床研究阶段，Teliso-V已获得美国食品药品监督管理局（Food and Drug Administration, FDA）突破性认定，用于含铂治疗后进展的晚期MET阳性、EGFR野生型NSCLC患者^[63]^。Ib期研究入组了20例经MET-TKIs治疗进展的患者，疗效尚在观察中。

### 4.2 局部或其他全身治疗策略

在临床应用中，部分靶向治疗患者的进展表现为寡进展。可尝试在继续接受MET-TKIs治疗的基础上联合局部治疗^[64]^。寡进展通常被定义为出现在一处或有限数量器官的小病灶转移，且患者整体病情控制稳定，反映肿瘤存在异质性^[65]^。已有临床研究^[66,67]^证实立体定向放疗用于寡进展的驱动基因阳性的NSCLC患者可改善其PFS。对于孤立的脑实质转移或进展，继续MET-TKIs治疗基础上联合立体定向放疗或手术治疗也能够使患者获益。对于脑多个病灶转移或进展，可考虑全颅放疗，也可选择更高血脑屏障透过率的药物^[68]^。

25%-47%的患者无法经二次活检及NGS明确耐药机制，或虽可明确耐药机制但无对应靶向治疗方案。对于这部分患者，如出现明显症状、广泛的疾病进展，应进行含铂双药化疗、免疫治疗、抗血管治疗或联合治疗。MET变异发生率较低，相关报道较少。在EGFR-TKIs治疗后进展的患者中，细胞毒药物因杀伤肿瘤细胞的作用机制与TKIs治疗不同，能在TKIs耐药后发挥抗肿瘤作用^[69]^。相较于驱动基因阴性的NSCLC患者，携带METex14的NSCLC患者通常具有更高水平的程序性死亡配体1（programmed death ligand 1, PD-L1）表达。METex14患者中PD-L1高表达的比例为84%，而野生型患者PD-L1高表达比例为59%^[11]^。但对于MET-TKIs耐药NSCLC患者而言，单纯应用免疫检查点抑制剂（immune checkpoint inhibitors, ICIs）疗效有限，可考虑免疫联合化疗及抗血管治疗^[70]^。对于ICIs与MET-TKIs联合治疗的安全性及有效性仍需要进一步探讨^[33]^。图2展示了MET-TKIs耐药后的治疗选择。

**图2:**
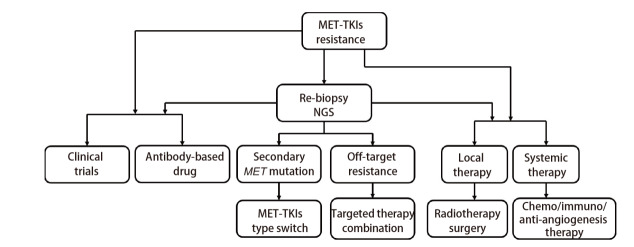
MET-TKIs耐药后治疗决策的流程图

## 5 总结与展望

MET突变是具有靶向治疗价值的致癌驱动基因，随着近年来的临床前和临床研究已经证实了HGF-MET通路在多种恶性肿瘤，特别是NSCLC中的重要性及治疗潜力。但MET-TKIs靶向治疗后发生获得性耐药是不可避免的。近年来分子诊断技术不断发展，MET-TKIs治疗失败后可能的耐药分子机制已进行了初步的探索，如MET点突变、METamp、旁路途径激活等导致耐药，仍有部分患者无法通过分子诊断明确耐药机制。对于MET-TKIs耐药后的治疗策略，需要对患者的体能状态（performance status, PS）评分、进展状态、耐药机制、既往治疗经过及安全性等方面进行综合考量。目前可选择的治疗策略包括更换MET-TKIs类型、靶向联合治疗、局部治疗、化学治疗或±ICIs、±抗血管等治疗模式。寻求临床研究对MET-TKIs耐药的患者也是一个很好的选择。新一代的MET-TKIs研发同样重要，通过分子诊断明确耐药机制并对TKIs类药物进行更新，改善患者临床获益。同时也期待MET-ADC、双特异性抗体等其他治疗药物有新的临床研究数据报道。

Competing interests

The authors declare that they have no competing interests.
